# Update on Hereditary Kidney Stone Disease and Introduction of a New Clinical Patient Registry in Germany

**DOI:** 10.3389/fped.2018.00047

**Published:** 2018-03-07

**Authors:** Jan Halbritter, Anna Seidel, Luise Müller, Ria Schönauer, Bernd Hoppe

**Affiliations:** ^1^Division of Nephrology, Department of Internal Medicine, University of Leipzig, Leipzig, Germany; ^2^Division of Pediatric Nephrology, University Children’s Hospital, Bonn, Germany

**Keywords:** nephrolithiasis, hereditary, nephrocalcinosis, kidney stone disease, monogenic, registry

## Abstract

Kidney stone disease is an increasingly prevalent condition with remarkable clinical heterogeneity, with regards to stone composition, age of manifestation, rate of recurrence, and impairment of kidney function. Calcium-based kidney stones account for the vast majority of cases, but their etiology is poorly understood, notably their genetic drivers. As recent studies indicate, hereditary conditions are most likely underestimated in prevalence, and new disease genes are constantly being identified. As a consequence, there is an urgent need of a more efficient documentation and collection of cases with underlying hereditary conditions, to better understand shared phenotypic presentation and common molecular mechanisms. By implementation of a centralized patient registry on hereditary kidney stone disease in Germany, we aim to help closing the vast knowledge gap on genetics of kidney stone disease. In this context, clinical registries are indispensable for several reasons: first, delineating better phenotype–genotype associations will allow more precise patient stratification in future clinical research studies. Second, identifying new disease genes and new mechanisms will further reduce the rate of unknown nephrolithiasis/nephrocalcinosis etiology; and third, deciphering new molecular targets will pave the way to develop drugs for recurrence prevention in severely affected families.

Incidence and prevalence of kidney stone disease continues to rise in the general population. With a lifetime prevalence of up to 10%, nephrolithiasis (NL) and nephrocalcinosis (NC) are therefore major health burdens, especially in the Western World (1). NL and NC are associated with significant morbidity and progression to chronic kidney disease due to recurrence, repetitive surgical/endoscopic intervention, and concomitant inflammation. On a simplified level, kidney stone formation results from an imbalance of urinary inhibitors (e.g., citrate, magnesium, uromoduline, and pyrophosphate) and promoters (e.g., oxalate, calcium, phosphate, urate, and cystine) of crystallization, exceeding supersaturation with consecutive aggregation, nucleation, and stone growth at Randall’s plaque (Figure 1). This imbalance can be due to altered enteral and/or renal handling of either promotors or inhibitors, such as enteral malsecretion of oxalate or renal malreabsorption of calcium (Figure 1).

**Figure 1:**
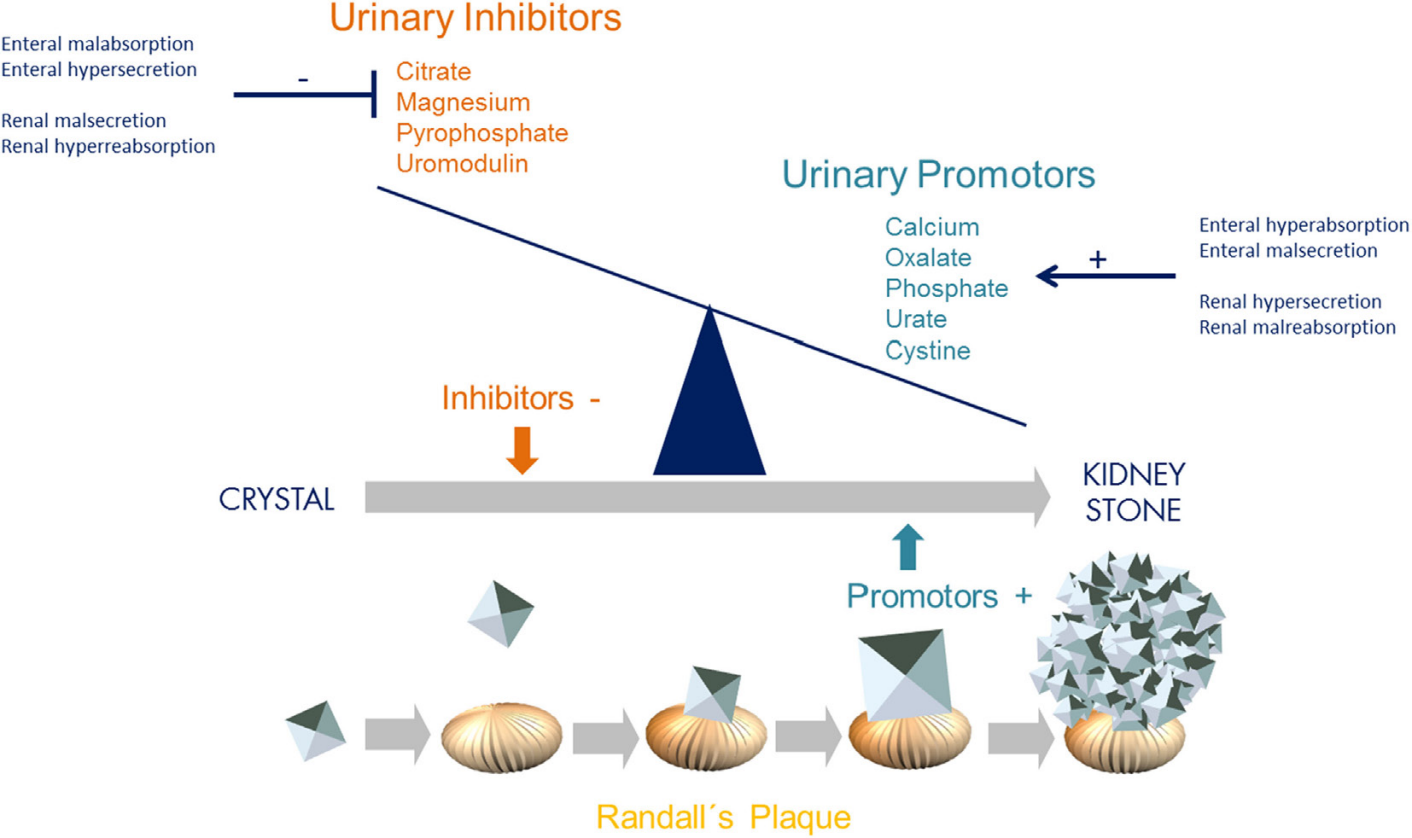
Imbalance of urinary inhibitors and promotors of crystallization leading to kidney stone formation. Concentration of urinary inhibitors and promotors is influenced and controlled by both intestinal and renal transporters (GI–kidney axis). These transporters and exchangers, such as SLC26A1, are responsible for secretion and absorption. With regards to oxalate, enteral hyperabsorption but malsecretion, and/or renal hypersecretion but malabsorption leads to urinary oxalate levels exceeding supersaturation and thereby promoting crystallization and consecutive stone formation [adapted from Ref. (2)].

The underlying etiology of NL is thought to be multifactorial with an environmental, notable dietary, hormonal, and genetic component. In twin studies, the heritability of kidney stones has been estimated at 56% (3), and up to two-thirds of hypercalciuric stone formers have relatives with NL (4). Although calcium-containing kidney stones account for more than 80% of all, the genetic basis of such stones remains largely unknown (5). Except for variants in *CLDN14, TRPV5, SLC34A1, ALPL, CASR*, and *UMOD*, genome-wide association studies have yet to yield substantial genetic factors (6–8). However, risk alleles have been identified within genes that were also found to transmit the disease on a Mendelian basis, such as *CASR, SLC34A1*, and *SLC2A9* (9, 10). To date, more than 30 single genes with an Online Mendelian Inheritance in Man-defined phenotype have been identified to be implicated in NL/NC, if mutated (Table 1).

**Table 1:** Genes, known to cause monogenic forms of NL/NC.

Gene symbol^a^	Gene name	Disease entity	MIM phenotype #	Mode	Reference
*ADCY10*	Adenylate cyclase 10	Idiopathic (absorptive) hypercalciuria, susceptibility	143870	AD	(11)
*AGXT*	Alanine-glyoxylate aminotransferase	Primary hyperoxaluria (PH), type 1, PH1	259900	AR	(12)
*APRT*	Adenine phosphoribosyltransferase	Adenine phosphoribosyltransferase deficiency, APRT	614723	AR	(13)
*ATP6V0A4*	ATPase, H+ transporting, lysosomal V0 subunit a4	dRTA	602722	AR	(14)
*ATP6V1B1*	ATPase, H+ transporting, lysosomal, V1 subunit B1	dRTA with deafness	267300	AR	(15)
*CA2*	Carbonic anhydrase II	Osteopetrosis + d/pRTA	259730	AR	(16)
*CASR*	Calcium-sensing receptor	Hypocalcemia with Bartter syndrome/hypocalcemia, AD	601198	AD	(17)
*CLCN5*	Chloride channel, voltage-sensitive 5	Dent disease/NL, type 1	300009/310468	XR	(18)
*CLCNKB*	Chloride channel, voltage-sensitive Kb	Bartter syndrome, type 3	607364	AR	(19)
*CLDN16*	Claudin 16	Familial hypomagnesemia with hypercalciuria and NC, FHHNC	248250	AR	(20)
*CLDN19*	Claudin 19	Familial hypomagnesemia with hypercalciuria and NC with ocular abnormalities	248190	AR	(21)
*CYP24A1*	Cytochrome P450, family 24, subfamily A, polypeptide 1	1,25-(OH) D-24 hydroxylase deficiency, infantile hypercalcemia	143880	AR	(22)
*FAM20A*	Family with sequence similarity 20, member A	Enamel renal syndrome, amelogenesis imperfect, and NC	204690	AR	(23)
*GRHPR*	Glyoxylate reductase/hydroxypyruvate reductase	PH, type 2, PH2	260000	AR	(24)
*HNF4A*	Hepatocyte nuclear factor 4, alpha	MODY + Fanconi syndrome + NC (p.R76W)	125850	AD	(25)
*HOGA1*	4-Hydroxy-2-oxoglutarate aldolase 1	PH, type 3, PH3	613616	AR	(26)
*HPRT1*	Hypoxanthine phosphoribosyltransferase 1	Kelley–Seegmiller syndrome, partial HPRT deficiency, HPRT-related gout	300323	XR	(27)
*KCNJ1*	Potassium inwardly rectifying channel, subfamily J, member 1	Bartter syndrome, type 2	241200	AR	(28)
*MAGED2*	Melanoma antigen, family D, 2	Bartter syndrome, type 5	300971	XR	(29)
*OCRL*	Oculocerebrorenal syndrome of Lowe	Lowe syndrome/Dent disease 2	309000/300555	XR	(30)
*SLC12A1*	Solute carrier family 12, member 1	Bartter syndrome, type 1	601678	AR	(31)
*SLC26A1*	Solute carrier family 26 (sulfate transporter), member 1	Ca-oxalate-NL	167030	AR	(32)
*SLC22A12*	Solute carrier family 22 (organic anion/urate transporter), member 12	Renal hypouricemia, RHUC1	220150	AD/AR	(33)
*SLC2A9*	Solute carrier family 2 (facilitated glucose transporter), member 9	Renal hypouricemia, RHUC2	612076	AD/AR	(10)
*SLC34A1*	Solute carrier family 34 (sodium phosphate), member 1	Hypophosphatemic NL, osteoporosis-1, NPHLOP1/Fanconi renotubular syndrome 2	612286/613388	AD/AR	(34)
*SLC34A3*	Solute carrier family 34 (sodium phosphate), member 3	Hypophosphatemic rickets with hypercalciuria	241530	AR	(35)
*SLC3A1*	Solute carrier family 3 (cystine, dibasic and neutral amino acid transporters, activator of cystine, dibasic and neutral amino acid transport), member 1	Cystinuria, type A	220100	AR	(36)
*SLC4A1*	Solute carrier family 4, anion exchanger, member 1	Primary dRTA, dominant/recessive	179800/611590	AD/AR	(37)
*SLC7A9*	Solute carrier family 7 (glycoprotein-associated amino acid transporter light chain, bo, +system), member 9	Cystinuria, type B	220100	AD/AR	(38)
*SLC9A3R1*	Solute carrier family 9, subfamily A (NHE3, cation proton antiporter 3), member 3 regulator 1	Hypophosphatemic NL, osteoporosis-2, NPHLOP2	612287	AD	(39)
*VDR*	Vitamin D (1,25-dihydroxyvitamin D3) receptor	Idiopathic hypercalciuria	277440	AD	(40)
*XDH*	Xanthine dehydrogenase	Xanthinuria, type 1	278300	AR	(41)

*^a^For HNF4A the MIM-phenotype number denotes MODY type 1, as occurrence of Fanconi syndrome and NC has only been shown in the presence of a specific allele: p.R76W*.

*AD, autosomal dominant; AR, autosomal recessive; dRTA, distal renal tubular acidosis; MODY, maturity onset diabetes of the young; NC, nephrocalcinosis; NL, nephrolithiasis; pRTA, proximal renal tubular acidosis; XR, X-chromosomal recessive*.

Modes of inheritance in monogenic forms include autosomal-dominant, autosomal-recessive, and X-linked transmission. Interestingly, in several of these genes, both recessive and dominant modes of inheritance have been reported: *SLC7A9, SLC34A1, SLC34A3, SLC2A9, SLC22A12*, and *SLC4A1*. While most of the syndromic and severe congenital disorders exhibit a recessive inheritance pattern (Bartter, Lowe, Dent, FHHNC, and distal renal tubular acidosis with sensorineural deafness), milder conditions are rather associated with mutations in dominant genes. The majority of encoded proteins constitute renal solute transporters (e.g., SLC34A1, SLC34A3, and SLC9A3R1), but also chloride channels (CLCN5), tight-junction proteins (e.g., CLDN16/CLDN19), and metabolizing enzymes (e.g., AGXT, APRT, and CYP24A1) have been found defective in patients with NL/NC. Hence, the underlying defect is mostly located in the tubular system of the kidney itself and can therefore be attributed as tubulopathy. Conversely, *a priori* extrarenal conditions, as in primary hyperoxaluria (PH) where dysfunction of liver enzymes (AGXT, GRHPR, and HOGA1) cause oxalate accumulation with secondary renal affection, are conceivable causes of NL/NC. Although each disease phenotype is thought to represent a relatively rare entity, single-gene causes may account for a significant number of patients by their broad genetic heterogeneity (42). Apart from genetic heterogeneity, there is also an allelic variation, where truncating variants rather result in a loss of function and missense variants (hypomorphs) may cause rather subtle defects, which can be clinically overseen, especially in adult stone formers. Another recently appreciated phenomenon is about gene dosage effects in several of the aforementioned kidney stone genes. In *SLC34A3* for instance, encoding one of the main phosphate transporters in the proximal tubule (NaPiIIc), it was shown that heterozygous individuals can no longer be merely regarded as healthy carriers, as they display renal calcifications and/or bone manifestation significantly more frequent than wild-type individuals; but still to a lesser degree than biallelic (homozygous and compound heterozygous) individuals (43). Similar observations were reported for families with mutations in *CYP24A1* (44). The contribution of monogenic disorders to the overall prevalence of kidney stone disease has not been studied comprehensively in the past. Especially, genetic evidence based on broad screenings of a multitude of causative genes in large patient cohorts is lacking. Comprehensive genetic testing has been too costly and inefficient in the past. For most individuals with NL/NC, mutation analysis for a causative genetic defect has therefore not been accessible, despite the fact that knowledge of the molecular cause of NL/NC may have important consequences for prognosis, prophylaxis and/or treatment. Only rough estimates have been derived from clinical observation studies: based on a huge data collection of stone composition analysis, it was concluded that monogenic causes do not exceed 9.6% in children and 1.6% in adults (45). In the last decade, however, this situation has begun to change, with the advent of high-throughput sequencing techniques.

## High-Throughput Mutation Analysis in Patients with NL/NC

To investigate patients with kidney stone disease for the presence of pathogenic mutations in known disease genes, we established a gene panel based on microfluidic multiplex-PCR and consecutive NextGen sequencing (Fluidigm™/NGS) (46, 47).

**Figure 2:**
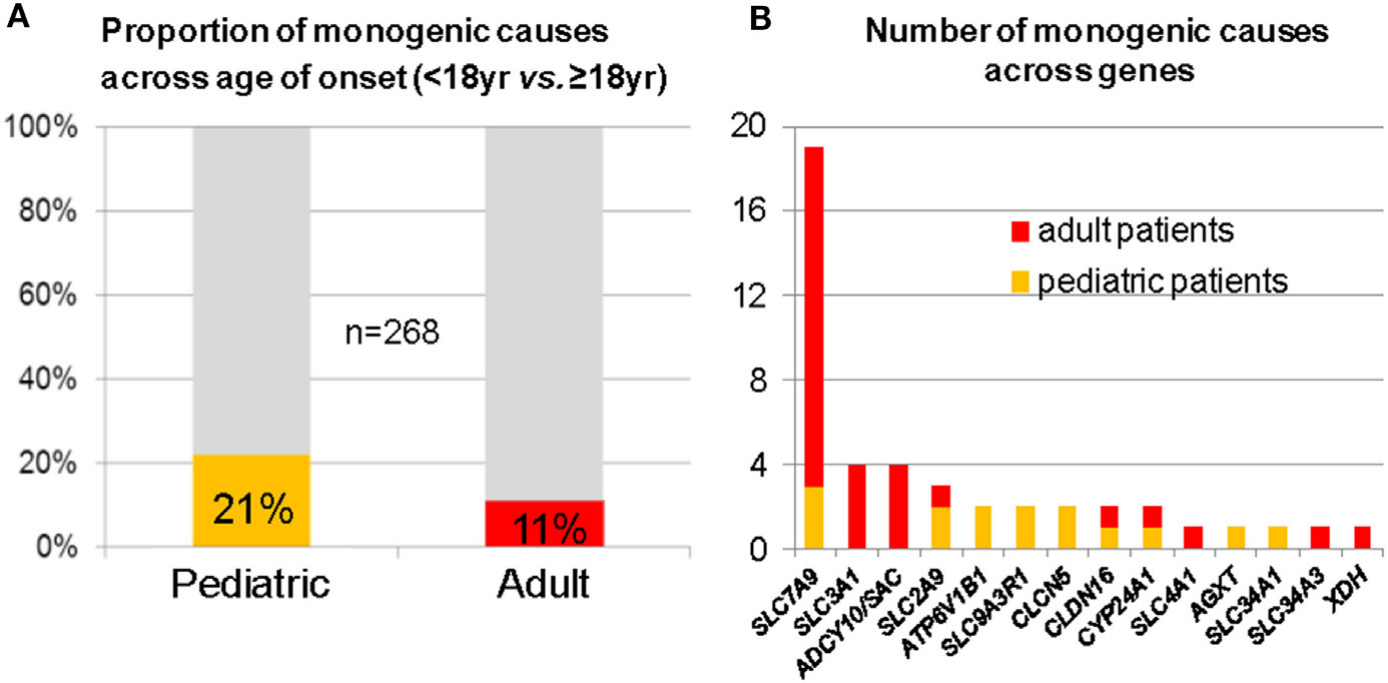
Mutation analysis of 30 known monogenic nephrolithiasis (NL)/nephrocalcinosis (NC) genes in 268 patients with NL/NC. **(A)** Fraction of monogenic causes in pediatric and adult subcohort. **(B)** Number of monogenic causes across genes (red denotes adults; orange denotes pediatric patients). Of note, *SLC7A9* was found most frequently mutated, especially in young adults.

In a “pilot-study,” we consecutively recruited 268 genetically unresolved individuals from typical kidney stone clinics; 102 pediatric and 166 adult probands. As a result, we identified 50 deleterious variants in 14 out of 30 analyzed genes, leading to a molecular diagnosis in 15% of all cases. In the pediatric subgroup, we detected a causative mutation in 21%, while among adults, deleterious variants were present in 11% (Figure 2A) (48). Mutations in the cystinuria-gene *SLC7A9* were found most frequently in the adult cohort (Figure 2B). Two follow-up studies were able to confirm these results. First, in an exclusively pediatric cohort of 143 NL/NC patients, 17% of cases were explained by mutations in 14 different genes (49). Second, in a cohort of 51 families with age of NL/NC manifestation before 25 years, targeted WES was used to detect a genetic cause in almost 30% (50). Not surprisingly, recessive mutations were more frequently found among neonates and in cases of congenital disease, whereas dominant conditions usually manifested later in life. These data indicate that genetic kidney stone disease is an underdiagnosed condition, despite the fact that the molecular diagnosis will potentially influence prognosis, prophylaxis, and/or treatment. A limitation worth mentioning, however, is a potential selection bias due to recruitment from specialist kidney stone clinics in all of the three aforementioned studies.

## Identification of Novel Human Disease Genes by Candidate-Gene Approach

High-throughput mutation analysis is also used to screen for pathogenic variants in various candidate genes. One of the most interesting recent findings was the discovery of human mutations in *SLC26A1* (32). Since the first description of Ca-oxalate (CaOx) kidney stone formation and NC in Slc26a1 (Sat1)-knockout mice by Dawson et al. in 2010, *SLC26A1* has been a bona fide NL-candidate gene (51). SLC26A1 encodes an anion exchanger expressed at the basolateral membrane of proximal renal tubules, ileum, and jejunum. Consequently, by using a candidate-gene approach, pathogenic variants were identified in humans with a history of early onset CaOx-NL, namely, two unrelated individuals with biallelic missense variants (32). Functionally, pathogenicity of the identified variants was demonstrated *in vitro*, leading to intracellular mis-trafficking and impaired transport activity (32). Defective SLC26A1 therefore constitutes a new cause of CaOx-NL and should be considered when testing individuals for causes of recurrent CaOx-stone formation.

## New Clinical Patient Registry for Hereditary Kidney Stone Disease

Most epidemiological data on increasing prevalence in Western countries are derived from US databases. Although urgently needed, centralized European databases are not available at the time. As aforementioned genetic studies on prevalence of hereditary kidney stone disease were executed with small cohorts from specialized centers in both Europe and the US, a translation to the general situation in Europe is not valid. While in the US, the *Rare Kidney Stone Consortium* constitutes a platform that integrates and coordinates registry, basic science, and clinical research activities for rare conditions such as cystinuria, PH, APRT deficiency, Dent and Lowe disease, no comparable data collection on patients with hereditary kidney stone disease has been implemented neither in Europe nor in Germany today. In collaboration with the existing European PH registry, *OxalEurope* (Prof. Bernd Hoppe, University of Bonn), and through funding by *Deutsche Forschungsgemeinschaft* and *Else Kröner-Fresenius Stiftung*, we recently established a clinical patient “Registry for hereditary kidney stone disease” at the University of Leipzig. The registry is nationally supported by the German Societies of Adult Nephrology (DG*f*N) and Pediatric Nephrology (GPN). It is further enrolled at the German Clinical Trials Register (DRKS-ID: DRKS00012891). As a fundamental part of study recruitment, high-throughput mutation analysis for known and novel kidney stone genes is offered on a research basis for patients without an established molecular diagnosis but with a clinical picture that points to an underlying genetic susceptibility: e.g., early age of onset (<40 years), positive family history, indicative phenotypes such as NC, cystinuria, or RTA, and severely recurrent NL (>3×) (Table 2). While patients with an already established genetic diagnosis are generally enrolled, cases with secondary NL/NC causes, such as malignancy, sarcoidosis, and primary hyperparathyroidism, do not get included in genetic analysis. To actively enroll patients, a clinical center will usually need approval by the local Institutional Review Board; a process for which we offer our help and assistance by providing respective templates. Upon ethics approval, consent form and clinical data sheets (e.g., clinical questionnaire) can be downloaded from our registry website (http://www.mks-registry.net). To ensure thorough clinical phenotyping, we will be asking for substantial patient information such as ethnicity, consanguinity, family history, age of onset, recurrence (defined as every putatively new kidney stone event), daily fluid intake, surgical interventions, and extrarenal involvement among others. The documents can be filled in by the patient with the help of the enrolling physician. In addition, biochemical serum parameters, including creatinine, eGFR, PTH, vitamin D, electrolytes, uric acid, and urinalysis (pH, calcium, phosphate, magnesium, uric acid, citrate, oxalate, and cystine, preferably from 24-h urine, if not spot urine), as well as data on stone composition analysis will be requested upon enrollment. Taking into account that 24-h urine collection and stone composition analysis is not routinely performed at all institutions, we include these parameters upon availability. 2-yearly clinical follow-up visits of enrolled patients are desirable but not mandatory. After registration, recruiting clinical centers will be provided with a personalized login to enter patient data *via* our registry website (http://www.mks-registry.net). Alternatively, we offer entering the data electronically when sent to us on paper. Entered data will be stored on a secured server and can be accessed by participating clinical centers to view their own patient data. The following websites provide further information:

https://www.dgfn.eu/hereditaere-nierensteinleiden.html
https://www.drks.de/drks_web/navigate.do?navigationId=trial.HTML&TRIAL_ID=DRKS00012891

**Table 2:** Inclusion criteria for mutation analysis in clinical patient registry.

Clinical criteria
Pediatric age of onset or onset during early adulthood (<40 years) plus
Positive family history or
Recurrence (>3×) or
Indicative phenotype (e.g., RTA, cystinuria, and NC) or
Established molecular genetic diagnosis

In summary, kidney stone disease is an increasingly prevalent condition which is clinically heterogeneous and poorly understood, notably its genetic drivers. As a series of recent studies indicated, monogenic conditions are most likely underestimated in prevalence. By implementation of a centralized patient registry on hereditary kidney stone disease, we will contribute to overcome, at least in part, the vast knowledge gap on genetics of kidney stone disease. In this context, clinical registries are valuable sources for several reasons: first, delineating better phenotype–genotype associations will be crucial for more precise patient stratification in future clinical research studies. Second, identifying new disease genes with new disease mechanisms will diminish the gap of unknown NL/NC etiology; and third, deciphering new molecular targets helps to pave the way for developing drugs of recurrence prevention in severely affected families.

## Ethics Statement

This study was carried out in accordance with the recommendations of “Ethikkommission an der Medizinischen Fakultät der Universität Leipzig” with written informed consent from all subjects. All subjects gave written informed consent in accordance with the Declaration of Helsinki. The protocol was approved by the “Ethikkommission an der Medizinischen Fakultät der Universität Leipzig.”

## Author Contributions

JH conceived and wrote the manuscript. BH, AS, LM, and RS edited the manuscript and built up the registry’s infrastructure that is introduced to the reader.

## Conflict of Interest Statement

The authors declare that the research was conducted in the absence of any commercial or financial relationships that could be construed as a potential conflict of interest.
